# The response of microbial necromass C and its contribution to SOC to vinasse biochar based on a pot experiment

**DOI:** 10.3389/fmicb.2026.1737822

**Published:** 2026-06-17

**Authors:** Wenjing Chen, Kelu Chen, Bi Chen, Yang Wu

**Affiliations:** 1 Moutai Institute, Renhuai, Guizhou, China; 2 Guizhou Province Engineering Research Center for Efficient Utilization of Distiller’s Grains, Renhuai, Guizhou, China; 3 Chishui River Middle Basin, Watershed Ecosystem, Observation and Research Station of Guizhou Province, Renhuai, Guizhou, China; 4 Academy of Science and Technology, Kweichow Moutai Co., Ltd., Renhuai, Guizhou, China; 5 Northwest Agriculture and Forestry University, Yangling, Shaanxi, China

**Keywords:** biochar, microbial necromass carbon, pyrolysis temperatures, sorghum, vinasse

## Abstract

**Introduction:**

Microbial necromass carbon (MNC) is a cornerstone of stable soil organic carbon (SOC), yet how biochar amendments impact its dynamics over time remains poorly understood.

**Methods:**

Here, we investigated the temporal response of MNC in a sorghum-cultivated soil amended with vinasse biochar produced at four pyrolysis temperatures (350–650 °C) over a 120-day experiment using amino sugar biomarkers and microbial profiling.

**Results:**

We reveal a distinct time-dependent biphasic response. Initially (days 30 and 60), biochar application suppressed both fungal and bacterial MNC, attributed to a nutrient-driven “priming effect” and increased microbial turnover, which accelerated decomposition. Notably, higher pyrolysis temperatures attenuated this initial loss. However, a critical reversal occurred by day 120, where all biochar treatments consistently increased MNC stocks, independent of pyrolysis temperature. This shift likely reflects the attenuation of priming and the onset of stabilization processes like aggregate formation.

**Discussion:**

Consequently, the initial negative contribution of necromass to total SOC diminished over time. These findings highlight that short-term assessments may misjudge biochar’s potential for stabilizing microbial carbon. Optimizing vinasse biochar strategies requires balancing temporary priming risks against eventual long-term MNC accumulation.

## 1 Introduction

Enhancing soil organic carbon (SOC) through management practices is an effective strategy to mitigate global warming (Mandal et al., 2022). For decades, the SOC pool has traditionally been viewed as originating primarily from decomposing plant litter (Lehmann and Kleber, 2015; Kallenbach et al., 2016). However, a new paradigm emphasizes that microbial-derived compounds are not merely contributors to SOC, but are fundamental to its long-term stabilization (Zhu et al., 2020; Fan et al., 2021; Zhang X. et al., 2021). Microbial necromass—the remnants of dead microbial cells—serves as a primary source of proteins and lipids that, through interactions with soil minerals and physical protection within aggregates, form the persistent core of stable SOC (Yuan et al., 2021; Cai et al., 2023). In fact, current estimates indicate that these microbial residues can constitute up to 50% of the total SOC, highlighting that their contribution to carbon storage has been significantly underestimated (Yuan et al., 2021). Therefore, given that microbial necromass carbon (MNC) constitutes a dominant fraction of stable SOC, understanding the factors controlling its accumulation is a prerequisite for developing effective sequestration strategies.

The application of biochar is a widely recognized method for enhancing SOC (Maroušek et al., 2019; Liao et al., 2022). However, the specific efficacy of biochar in mediating the crucial MNC pool—rather than just total SOC—remains a subject of intense debate, with existing literature yielding complex and often contradictory results. This inconsistency largely stems from two critical knowledge gaps. First, current research largely relies on static snapshots, failing to capture the distinct temporal phases of biochar’s influence. Biochar application often induces an immediate “priming effect,” accelerating the decomposition of native organic matter (including existing necromass), while potentially fostering MNC accumulation over longer periods through habitat provision and substrate supply (Chen Y. et al., 2023). Without a high-resolution time-series analysis, it is impossible to resolve the net balance between these opposing short-term losses and long-term gains. Second, the mechanistic link between biochar’s production parameters and MNC dynamics remains poorly defined. Pyrolysis temperature is the master variable dictating biochar’s physicochemical properties, such as surface area, pH, and labile C content (Tomczyk et al., 2020). While it is known that these properties regulate microbial community structure and enzyme activity, how they systematically combine to drive the specific microbial turnover processes required for MNC accumulation is largely unknown. Addressing these gaps is critical for optimizing biochar design to maximize carbon sequestration.

Elucidating the microbial mechanisms linking pyrolysis temperature to MNC accumulation is particularly challenging because of the intricate feedbacks between microbial traits and soil conditions. Pyrolysis temperature regulates nutrient availability and stoichiometry, which in turn shifts microbial community composition and metabolic strategies (Chen et al., 2022; Deshoux et al., 2023). For instance, low-temperature biochar (<500 °C) typically enhances the activities of enzymes involved in C, N, and P acquisition, whereas high-temperature biochar (≥500 °C) often shows negligible effects on enzymatic activity (Liao et al., 2022) but may induce stronger carbon limitation due to high recalcitrance and nutrient retention (Guo et al., 2020). Furthermore, fungal richness and microbial biomass frequently show negative correlations with increasing pyrolysis temperature (Deshoux et al., 2023). Despite these isolated insights, a comprehensive understanding of how biochar-induced shifts in microbial traits (e.g., diversity, enzyme activity) explicitly translate into necromass retention efficiency over time remains limited.

To investigate these dynamics, we focused on the sorghum cropping system, a critical component of global agriculture and bioenergy production. Sorghum (*Sorghum bicolor* (L.) Moench) is the fifth most important cereal crop globally (Kazungu et al., 2023) and is vital for resource-poor farmers in arid regions. In China alone, the sorghum planting area expanded significantly from 1978 to 2018, reflecting its growing economic importance (Li et al., 2021). Beyond its role as a food source, sorghum is extensively used in the brewing industry, generating vast quantities of vinasse (distiller’s grains) as a byproduct. This presents both a waste management challenge and a resource opportunity (Wright, 2011; Cheng et al., 2023). Converting vinasse into biochar and returning it to sorghum fields represents a promising circular economy approach. While biochar is known to improve soil health in sorghum fields (Kelland et al., 2020; Zhou et al., 2021; Yang et al., 2023; Yang et al., 2024), he potential of vinasse biochar to enhance carbon stability via the microbial necromass pathway has not been systematically evaluated.

To address these knowledge gaps, we conducted a sorghum pot experiment to investigate the dynamic impacts of vinasse biochar produced at varying pyrolysis temperatures on soil MNC stocks and their contribution to total SOC. By integrating amino sugar biomarker analysis with microbial community profiling (16S and ITS sequencing), enzyme assays, and soil property measurements, this study aimed to: (1) quantify the temporal responses of MNC concentration and its sequestration efficiency within the sorghum-soil system to different biochar types, and (2) elucidate the key biotic or abiotic mechanisms driving MNC accumulation, specifically identifying how pyrolysis temperature modulates the microbial traits responsible for necromass formation and stabilization.

## 2 Materials and methods

### 2.1 Study site

This study was conducted at the experimental field of Moutai Institute in Luban Town, Renhuai City, Guizhou Province, China (27°44′15.44″N, 1106°20′1.70″E). The elevation is approximately 850 meters above sea level. The annual mean temperature is about 16.3 °C, with an average annual precipitation of around 800–1,000 mm. The soil in this region is primarily purplish soil (Zhao et al., 2023), which is equivalent to red soil.

### 2.2 Preparation of biochar

Raw vinasse was oven dried at 65 °C for 48 h to a constant weight, then crushed and sieved to a particle size of <0.25 mm. This particle size was chosen to ensure uniform heat transfer during pyrolysis and to create a product that could be mixed homogeneously into the soil. For each pyrolysis run, approximately 200 g of the prepared feedstock was spread evenly in stainless steel trays with tightly fitting lids. Pyrolysis was conducted in a Microwave Muffle Furnace. To create the required anaerobic (oxygen free) environment essential for pyrolysis and to prevent combustion, high purity nitrogen gas was purged through the furnace chamber at a flow rate of 250 L·h^−1^ for 30 min prior to and during heating. Volatile gases produced during pyrolysis were safely vented via an outlet port. Samples were heated to final temperatures of 350, 450, 550, and 650 °C at a rate of 5 °C min^−1^. The target temperature was held for 1 h. Afterward, the furnace was switched off, and the biochar was allowed to cool naturally to below 100 °C under the continuous nitrogen flow, a process that took approximately 5 h. Each biochar type (BC_350_, BC_450_, BC_550_, and BC_650_) was produced in one large batch, which was then combined and homogenized to ensure a consistent material was used for the entire experiment (Wang X. et al., 2015). The key properties of the biochars, including BET Surface Area, Single point adsorption total pore volume, ash content, pH, total carbon (TC), and total nitrogen (TN), are detailed in Supplementary Figure S1.

### 2.3 Experimental design

The experiment employed a completely randomized factorial design with two factors: biochar treatment (five levels) and sampling time (three levels). The treatments consisted of a control (CK, no biochar) and four vinasse biochar amendments produced at different pyrolysis temperatures: 350 °C (BC_350_), 450 °C (BC_450_), 550 °C (BC_550_), and 650 °C (BC_650_). Destructive sampling was conducted at days 30, 60, and 120 after planting. For each treatment at each time point, three independent replicate pots were prepared, totaling 45 pots. Each pot served as a unique experimental unit sampled only once.

The experiment was established in early April 2023 using plastic buckets (23.5 cm height, 29 cm upper diameter, 22 cm lower diameter) buried with rims level to the ground to expose them to ambient outdoor conditions. Each pot was filled with 10 kg of air-dried soil collected from the top 20 cm of a nearby agricultural field with a long history of sorghum cultivation; basic properties in Supplementary Figure S1. For biochar treatments, biochar was applied at a rate of 5.0% (w/w, dry weight basis; 500 g pot^−1^) and thoroughly mixed with the soil to ensure homogeneity. This high application rate was chosen to elicit a strong and detectable signal on soil microbial processes within the limited duration of a pot experiment, a common practice in mechanistic studies (Cayuela et al., 2014; Wang J. et al., 2015). We acknowledge this rate is higher than typical field applications and address its implications in the discussion.

Seeds of a local sorghum cultivar (‘Hongyingzi’) were planted and thinned to one seedling per pot 1 week after germination. Soil moisture was maintained at approximately 70% of field capacity throughout the 120-day experiment. Pots were weighed every 2 days and replenished with local tap water to maintain target weights, ensuring consistent moisture conditions across treatments. Sorghum seedlings were planted to establish an active rhizosphere environment, ensuring continuous carbon input via root exudates to drive microbial turnover. The primary focus of this study was on soil biogeochemical processes; therefore, while plants were maintained to simulate realistic soil conditions, crop yield and biomass parameters were not the primary endpoints of this investigation.

### 2.4 Soil sampling

Destructive soil sampling was conducted on days 30, 60, and 120 post-planting, corresponding to key sorghum growth stages: late tillering, heading/stem elongation, and grain filling/maturation, respectively. At each time point, the three designated replicate pots per treatment were sampled. From each pot, five soil cores were collected from the 0–10 cm depth using a sterilized stainless steel hand auger (5 cm inner diameter), combined into a single composite sample, and sieved through a 2-mm mesh. Each composite sample was immediately divided into three subsamples: one stored at −80 °C for microbial community analysis, one stored at 4 °C for enzyme assays (assessed within 1 week), and one air-dried for the determination of amino sugars and other physicochemical properties.

### 2.5 Soil analysis

#### 2.5.1 Soil properties

Soil properties, including total organic carbon (SOC), total nitrogen (TN), total phosphorus (TP), soil available phosphorus (SAP), ammonium (NH_4_^+^-N), and nitrate (NO_3_^−^-N), were analyzed using air dried samples. SOC was determined by H_2_SO_4_-K_2_Cr_2_O_7_ oxidation, TN by the Kjeldahl method, and SAP by the Olsen method. TP and SAP were measured colorimetrically using the molybdate ascorbic acid method with a UV-1800 spectrophotometer (Shimadzu, Japan). Ammonium and nitrate concentrations were analyzed with a flow injection analyzer (AutoAnalyzer, Bran+Luebbe GmbH, Norderstedt, Germany) following the procedures outlined by Bremner and Mulvaney (1982) and Nelson and Sommers (1996). Dissolved organic carbon and nitrogen (DOC and DON) were determined at 4 °C using a TOC analyzer (liqui TOC II, Elementar, Germany). Soil pH was measured with a pH meter using a 1:5 soil to water ratio (Metrohm 702, Metrohm Ltd., Herisau, Switzerland) (Chen W. et al., 2023).

#### 2.5.2 The characterization of biochar

Use scanning electron microscope (TESCAN CLARA Xplore 30) to observe the morphology and surface characteristics of biochar samples. Randomly select the outer surface area of the biochar sample, place it on a black background adhesive plate, adjust the field of view clarity, select the structurally intact area for photography, analyze and save. Weigh 100 mg of biochar sample with a particle size of 0.15 mm and measure the C and N content using LECOCNS2000 instrument. Biochar pH was measured with a pH meter using a 1:20 biochar to water ratio (Metrohm 702, Metrohm Ltd., Herisau, Switzerland) (Chen W. et al., 2023).

#### 2.5.3 Microbial enzyme activity

The activities of seven key soil extracellular enzymes: *β*-1,4-glucosidase (BG), cellobiohydrolase (CBH), N-acetyl-β-D-glucosaminidase (NAG), Leucine-ɑ-aminopeptidase (LAP), acid phosphatase (AP), Catalase (CAT), Polyphenol oxidase (PPO), which are essential for the cycling of carbon, nitrogen, and phosphorus, were measured using a fluorometric microplate enzyme assay technique (Pritsch et al., 2004; German et al., 2011). Enzyme assays were conducted on 96 well microplates with eight replicates for each sample. Detailed methods for extracellular enzyme determination have been previously described in our earlier publication.(Chen W. et al., 2023).

#### 2.5.4 Microbial DNA extraction, amplicon sequencing, and data processing

Microbial genomic DNA was extracted using the Isolation Kit from MO BIO Laboratories according to manufacturer instructions. Fungal identification was conducted by amplifying the ITS1 region with primers ITS1F/ITS2-2043R, and bacterial identification by amplifying the 16S rRNA region with primers 338F/806R (Yan et al., 2017; Yu et al., 2019). Detailed methods for ITS amplification are provided in our previous publication (Chen W. et al., 2023). Sequencing was carried out on the Illumina HiSeq 2,500 platform, producing 2 × 250 bp paired end reads.

To ensure the generation of high quality clean tags, sequences were filtered using QIIME quality control protocols as described by Bokulich et al. (2013). The UCHIME algorithm was employed to sort samples based on barcode sequences and remove chimeric sequences (Edgar et al., 2011). Sequences were then clustered using UPARSE software, with operational taxonomic units (OTUs) defined at a 97% similarity threshold. Taxonomic classification of bacterial and fungal representative sequences was performed against the SILVA database and the UNITE + INSDC frameworks, using the RDP Classifier, BLAST, and QIIME software (Chen W. et al., 2023).

#### 2.5.5 Amino sugars

Soil amino sugars were extracted and analyzed as biomarkers for microbial necromass, following the classic protocol described by Zhang and Amelung (1996). Briefly, air-dried soil samples were hydrolyzed with 6 M HCl at 105 °C for 8 h. The hydrolysates were purified, dried, and derivatized to form aldononitrile acetates. The derivatives were separated and quantified using a gas chromatograph (Agilent 6890A, USA) equipped with an HP-5 column and a flame ionization detector (FID). Myo-inositol was used as the internal standard.

The concentrations of fungal necromass carbon (FNC) and bacterial necromass carbon (BNC) were calculated based on the concentrations of glucosamine (GluN) and muramic acid (MurN), using the conversion factors proposed by Liang et al. (2019).


$$\begin{array}{l} \text{Fungal necromass}\;C\;(\mathrm{mg}\;{\mathrm{kg}}^{-1})\\ =(\text{GluN}/179.17–2\times \text{MurA}/251.23)\times 179.17\times 9. \end{array}$$

$$\text{Bacterial necromass}\;C\;(\mathrm{mg}\;{\mathrm{kg}}^{-1})=\text{MurN}\times 45.$$

Where the molecular weights of glucosamine (GluN) and muramic acid (MurN) are 179.2 and 251.23, respectively. The conversion ratios are 9 for fungal-derived GluN to fungal necromass C and 45 for MurN to bacterial necromass C (Chen et al., 2025).

Total necromass carbon (C) is the combined total of fungal and bacterial necromass carbon. The proportions of fungal and bacterial necromass carbon within the soil organic carbon (SOC) reflect their respective contributions to SOC. The calculation method is as follows: Microbial necromass contribution (%) = Microbial necromass C/SOC * 100.

### 2.6 Statistical analysis

Prior to statistical analyses, all data were tested for normality and homogeneity of variance using the Shapiro–Wilk test and Levene’s test, respectively. When necessary, data were log-transformed to meet the assumptions of parametric analyses (McDonald, 2014; Cai et al., 2023).

Differences in biochar properties, soil physicochemical properties, microbial necromass carbon (fungal, bacterial, and total), their contributions to soil organic carbon (SOC), microbial *α*-diversity indices, and extracellular enzyme activities among treatments at each sampling time were evaluated using one-way analysis of variance (ANOVA), followed by Duncan’s multiple range test. Statistical significance was determined at *p* < 0.05.

Four diversity indices (Shannon, Simpson, Chao1, and ACE) were calculated based on the operational taxonomic unit (OTU) table. Then microbial α-diversity was calculated as the average of the Z-scores of the four diversity indices for each sample. This averaging approach prevents any single index with large raw values from dominating the composite score and is a widely established method in ecological studies. Variations in bacterial and fungal community composition among treatments were assessed using principal coordinates analysis (PCoA) based on Bray–Curtis dissimilarity matrices at the OTU level. The relative abundances of bacterial and fungal communities at the phylum level were calculated and visualized using stacked bar charts. Permutational multivariate analysis of variance (PERMANOVA, 999 permutations) was performed to test the significance of treatment effects on community structure. The first two principal coordinate axes (PCoA1 and PCoA2), which explained the greatest proportion of variation in community composition, were extracted for subsequent correlation and modeling analyses.

Spearman’s rank correlation analysis was used to examine relationships among biochar properties, microbial community attributes, extracellular enzyme activities, microbial necromass carbon pools, and diversity indices. Mantel tests were conducted to assess the associations among microbial community composition (Bray–Curtis distance matrices) biochar properties and soil biochemical variables (including enzyme activities and necromass carbon fractions).

The relative importance of microbial community composition, *α*-diversity, and extracellular enzyme activities in explaining variations in fungal and bacterial necromass carbon was quantified using dominance analysis implemented in the “relaimpo” package in R (Grömping, 2007). All univariate statistical analyses were performed using IBM SPSS Statistics 20.0 (IBM Corp., Armonk, NY, USA), while multivariate analyses, Mantel tests, and dominance analysis were conducted in R software.

## 3 Result

### 3.1 The properties of biochar produced at different temperatures

**Figure 1:**
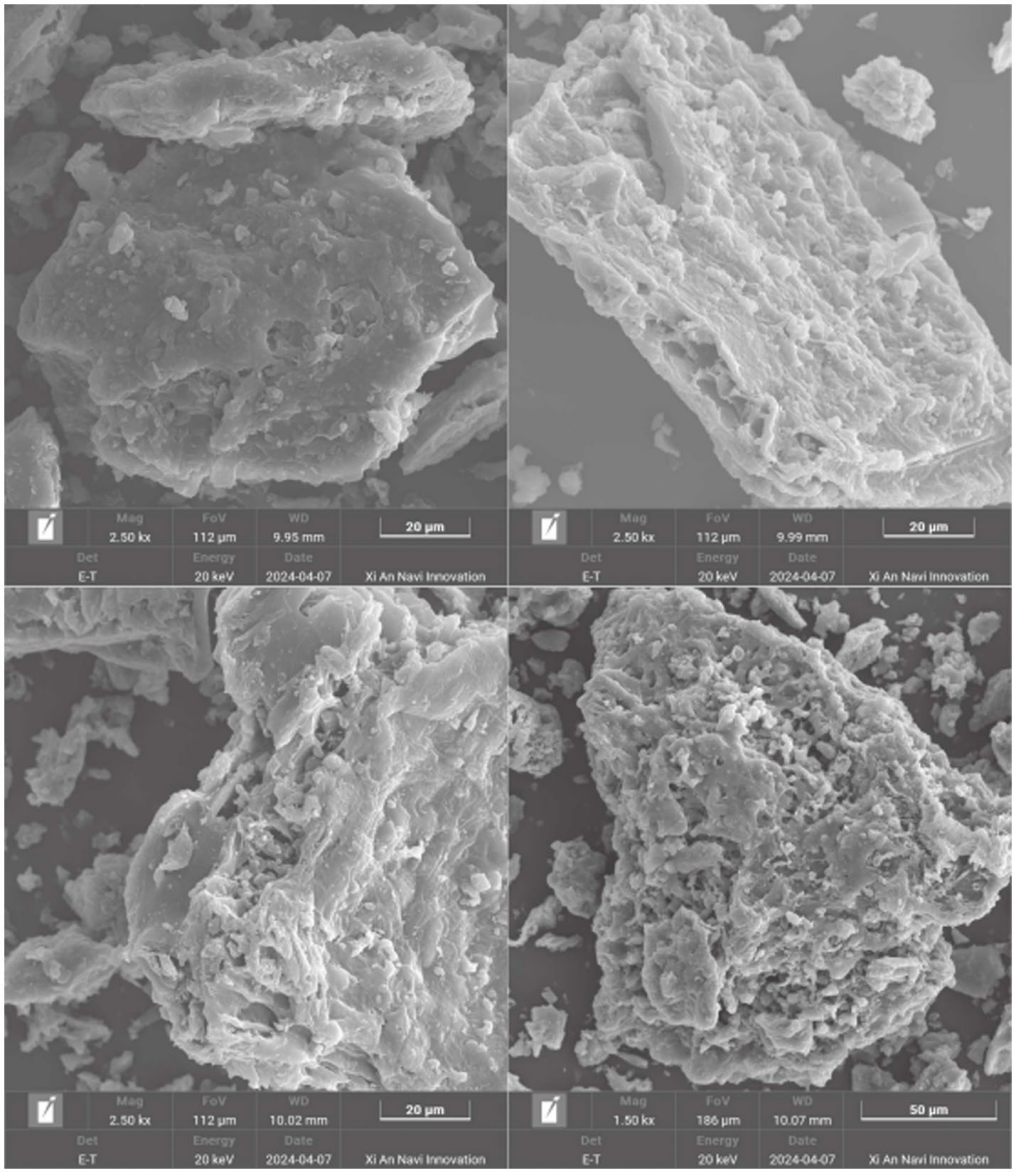
Scanning electron microscope (SEM) images illustrating the surface morphology and structure of vinasse biochar produced at different pyrolysis temperatures: (top left) 350 °C, (top right) 450 °C, (bottom left) 550 °C, and (bottom right) 650 °C.

The electron microscopy images of four types of biochar at different temperatures are shown in Figure 1. Through scanning electron microscopy images of distiller’s grains biochar, it can be seen that biochar from distiller’s grains at different temperatures has obvious pore structures, but the number and size of pores in biochar at different temperatures vary to some extent. At 350 °C, a clear pore structure appears on the surface of biochar, while at 450 °C, a clear pore structure appears inside biochar, with a small and irregular number of pores. When the temperature rises to 550 °C, the pore structure becomes denser and more regular, and at 650 °C, the pore structure is most dense.

Biochar properties varied significantly with increasing pyrolysis temperature (350–650 °C). Specifically, BET Surface Area and Single point adsorption total pore volume increased progressively with pyrolysis temperature, with the highest values observed in the 650 °C biochar (*p* < 0.05; Supplementary Figure S1). Similarly, ash content, total carbon (TC), and total nitrogen (TN) contents showed significant increases as pyrolysis temperature increased (*p* < 0.05). In addition, biochar pH increased consistently across the temperature gradient, indicating enhanced alkalinity at higher pyrolysis temperatures (*p* < 0.05).

### 3.2 Microbial necromass carbon and its contribution to SOC in response to different biochar produced at varying temperatures

A key finding was a biphasic, time dependent effect of biochar on microbial necromass carbon, with a general decrease observed in the early stages (days 30 and 60) followed by a significant increase in the later stage (day 120).

**Figure 2:**
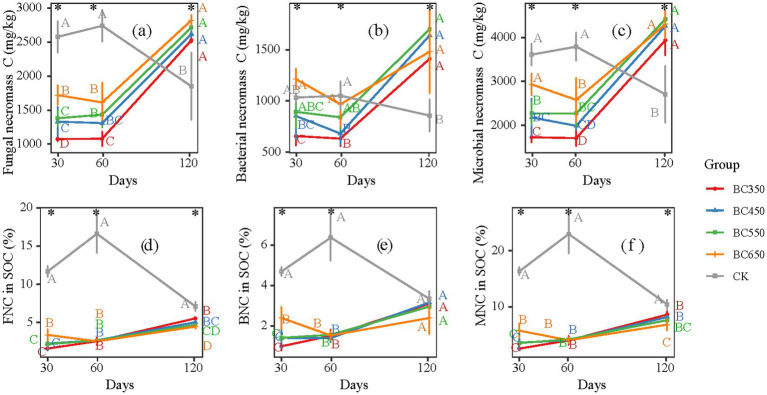
Concentrations of **(a)** fungal, **(b)** bacterial, and **(c)** total microbial necromass carbon, and **(d–f)** their respective contributions to soil organic carbon (SOC) across all treatments at days 30, 60, and 120. An asterisk (*) represents a significant main effect of treatment via ANOVA at that specific time point. Different capital letters indicate significant differences (*p* < 0.05) between different treatments within a time point (n = 3). FNC, fungal necromass carbon; BNC, bacterial necromass carbon; MNC, microbial necromass carbon; SOC, soil organic carbon; BC_350_, BC_450_, BC_550_, and BC_650_: biochar pyrolyzed at 350, 450, 550, and 650 °C, respectively; CK, control.

Initially, at day 30 and day 60, biochar decreased fungal necromass carbon (FNC) content compared to the control (CK). But only BC_350_ and BC_450_ decreased bacterial necromass carbon (BNC) compared to CK. During this early phase, higher pyrolysis temperatures generally resulted in higher MNC levels compared to lower temperature biochars. In contrast, by day 120, the trend had reversed completely. At this later stage, all biochar treatments significantly increased both fungal and bacterial necromass carbon compared to CK, independent of pyrolysis temperatures (Figure 2).

The contributions of fungal and bacterial necromass carbon to SOC were also significantly affected by treatment except for the BNC at 120 day (*p* < 0.05; Figure 2). In the early stages (days 30 and 60), the contribution of both fungal and bacterial necromass to SOC (FNC/SOC and BNC/SOC) was decreased by all biochar treatments. By day 120, however, while biochar treatments still showed a lower FNC/SOC ratio than CK, there was no longer a significant difference in the BNC/SOC ratio between biochar treatments and the control (Figure 2).

### 3.3 Soil microbial traits in response to different biochar produced at varying temperatures

**Figure 3:**
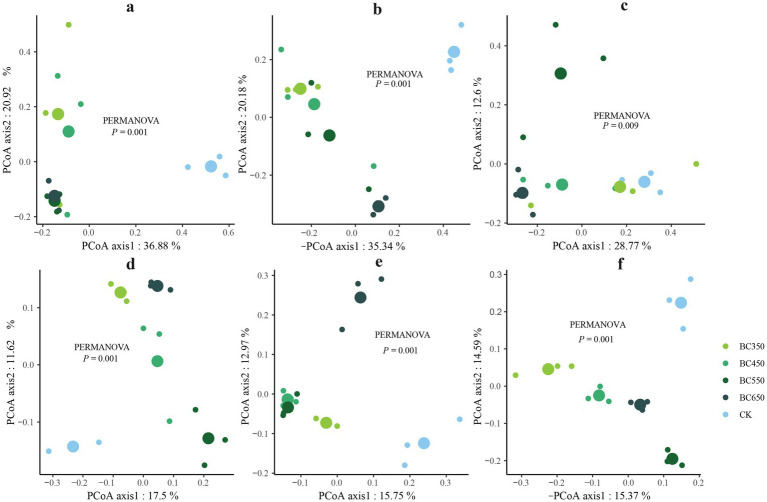
Principal coordinates analysis (PCoA) of soil fungal and bacterial community composition based on Bray-Curtis distances, showing the effects of different biochar treatments over time. The plots illustrate the shifts in **(a)** Fungal communities at day 30, **(b)** Fungal communities at day 60, **(c)** Fungal communities at day 120, **(d)** Bacterial communities at day 30, **(e)** Bacterial communities at day 60, and **(f)** Bacterial communities at day 120. Each point represents an individual biological replicate (*n* = 3). The proximity of points indicates the degree of similarity between microbial communities. The percentage of total variance explained by each principal coordinate axis is shown in parentheses. Permutational multivariate analysis of variance (PERMANOVA) indicated that community structures were significantly different among treatments at all time points (*p* < 0.05). CK, control (no biochar); BC_350_, BC_450_, BC_550_, and BC_650_: biochar pyrolyzed at 350, 450, 550, and 650 °C, respectively. Small circles represent individual biological replicates, and large circles represent the group centroids (mean values) for each treatment group.

For bacterial and fungal *α* diversities, only fungal α diversities on days 30 and 60 were affected by biochar treatment (Table 1). On day 30, biochar increased fungal α diversities, independent of pyrolysis temperature. On day 60, BC_450_, BC_550_, and BC_650_ increased fungal α diversities, while BC_350_ did not, and no significant differences were observed among the pyrolysis temperatures. The community composition of soil fungi and bacteria was significantly affected by all biochar treatments at all sampling times (*p* < 0.05; Figure 3). At the phylum level, *Actinobacteriota* in bacterial communities and *Ascomycota* and *Basidiomycota* in fungal communities exhibited significant responses to biochar addition (Supplementary Figure S2). Notably, the magnitude of these compositional shifts increased with rising biochar pyrolysis temperature, indicating a temperature-dependent effect of biochar on dominant microbial taxa.

**Table 1:** Alpha diversity indices of fungal and bacterial communities across all treatments at days 30, 60, and 120.

Microbial type	Treatment	Alpha diversity		
		Day 30	Day 60	Day 120
Fungi	CK	**−1.16 ± 0.04 B**	**−0.88 ± 0.37 B**	−0.69 ± 0.14 A
	BC_350_	**0.29 ± 0.57 A**	**−0.06 ± 0.27 AB**	−1.17 ± 0.81 A
	BC_450_	**0.54 ± 0.35 A**	**0.27 ± 0.44 A**	−0.02 ± 0.10 A
	BC_550_	**0.97 ± 0.07 A**	**0.35 ± 0.17 A**	−0.49 ± 0.27 A
	BC_650_	**0.96 ± 0.08 A**	**0.93 ± 0.18 A**	0.19 ± 0.10 A
Bacteria	CK	0.48 ± 0.54 AB	0.51 ± 0.28 A	1.01 ± 0.12 A
	BC_350_	−0.40 ± 0.38 AB	−0.06 ± 0.47 A	−0.31 ± 0.90 A
	BC_450_	0.67 ± 0.28 A	0.17 ± 0.37 A	0.04 ± 0.29 A
	BC_550_	−0.73 ± 0.46 B	−0.65 ± 0.43 A	−0.76 ± 0.37 A
	BC_650_	−0.16 ± 0.19 AB	−0.11 ± 0.56 A	0.31 ± 0.27 A

Bold font indicates a significant main effect of treatment at that specific sampling time (*p* < 0.05, *n* = 3). Different capital letters within a column (or time point) indicate significant differences between treatments (*p* < 0.05). Values represent mean ± SE.

**Figure 4:**
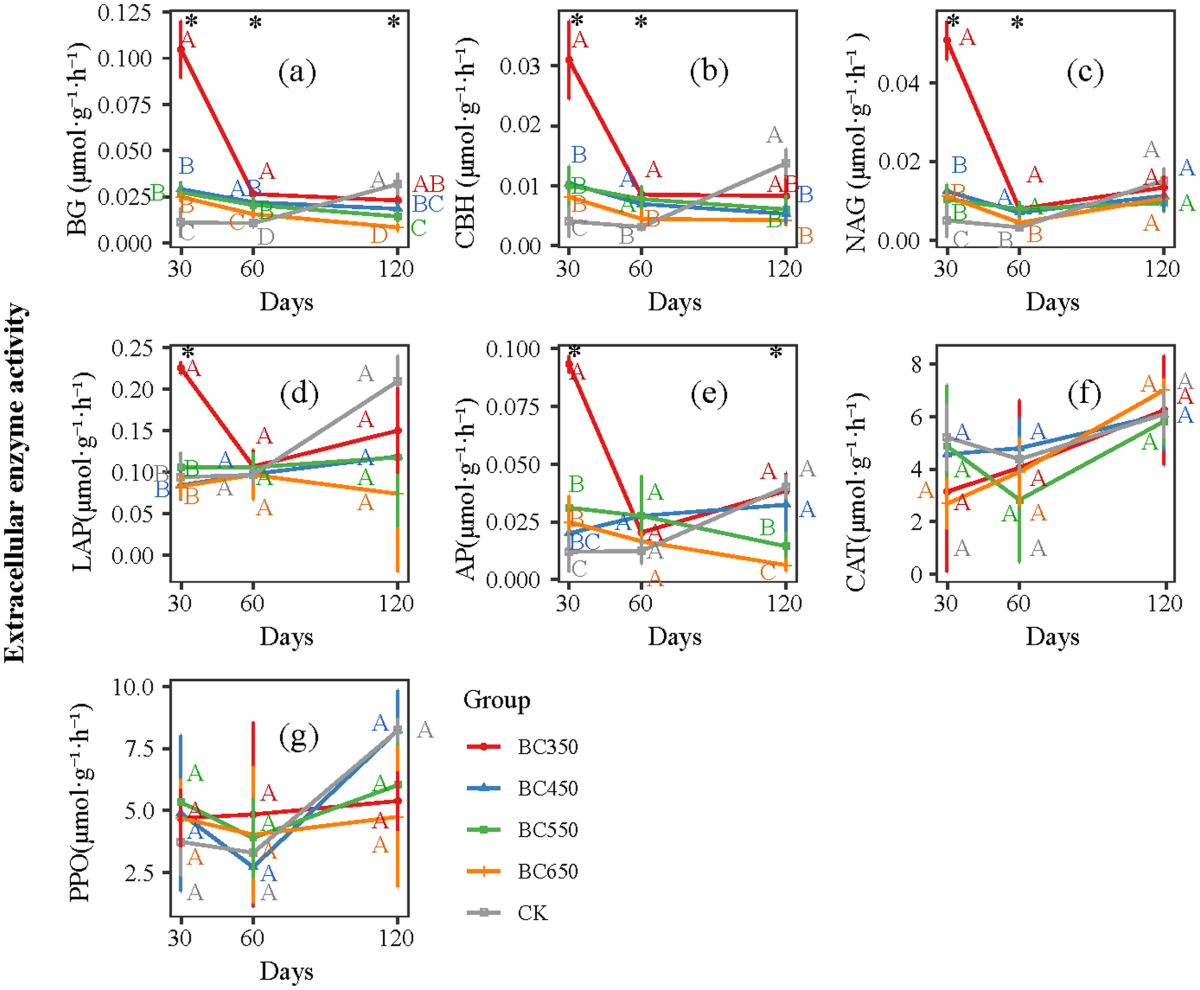
Microbial enzyme activities across all treatments at days 30, 60, and 120. An asterisk (*) represents a significant main effect of treatment via ANOVA at that specific time point. Different capital letters indicate significant differences (*p* < 0.05) between different treatments within a time point (*n* = 3). BG, β-1,4-glucosidase **(a)**; CBH, cellobiohydrolase **(b)**; NAG, N-acetyl-β-D-glucosaminidase **(c)**; LAP, leucine-ɑ-aminopeptidase **(d)**; AP, acid phosphatase **(e)**; CAT, catalase **(f)**; PPO, polyphenol oxidase **(g)**; BC350, BC450, BC550, and BC650: biochar pyrolyzed at 350 °C, 450 °C, 550 °C, and 650 °C, respectively; CK, control.

On day 30, BG, CBH, NAG, LAP, and AP activities were significantly altered by biochar treatments (*p* < 0.05). On day 60, BG, CBH, and NAG activities were significantly altered by biochar treatments (*p* < 0.05; Figure 4). On day 120, only BG, and AP activities were significantly altered by biochar treatments (*p* < 0.05). Specifically, on day 30, BC_550_, BC_450_ and BC_650_ increased BG, CBH and NAG activities compared to CK, and BC_350_ increased them compared to other treatments. Only BC_350_ increased LAP activity. The pattern of AP activity was similar to that of BG, CBH and NAG, but BC_450_ did not affected AP activity compared by CK. On day 60, BG activity was increased by biochar, and with increasing pyrolysis temperature, BG activity generally decreased. BC_350_, BC_450_, and BC_550_ increased CBH and NAG activities compared to CK and BC_650_. On day 120, BC_450_, BC_550_, and BC_650_ decreased BG activity compared to CK, and with increasing pyrolysis temperature, BG activity decreased. BC_450_, BC_550_, and BC_650_ decreased CBH, independent of pyrolysis temperatures. BC_550_ and BC_650_ decreased AP compared to other treatments (Figure 4).

### 3.4 Determinants of microbial traits and microbial necromass carbon under biochar treatment

Spearman correlation analysis revealed that on days 30 and 60, fungal *α* diversity was positively correlated with SOC, NH_4_^+^-N, NO_3_^−^-N, and pH, while bacterial α diversity was negatively correlated with pH. PCoA_1_ of the bacterial community was negatively correlated with DON. PCoA_2_ of the bacterial community was positively correlated with SOC, TP, DOC, NH_4_^+^-N, NO_3_^−^-N, SAP, and pH. PCoA_1_ of the fungal community was negatively correlated with NH_4_^+^-N. PCoA_2_ of the fungal community was negatively correlated with NO_3_^−^-N and pH (Table 2). On day 120, fungal α diversity was positively correlated with SOC, NH_4_^+^-N, and pH. Additionally, PCoA_1_ of the fungal community was negatively correlated with SOC, NH_4_^+^-N, NO_3_^−^-N, and pH, while PCoA_2_ of the bacterial community was negatively correlated with NO_3_^−^-N and pH. PCoA_1_ of the fungal community was negatively correlated with SOC and pH. PCoA_2_ of the fungal community was positively correlated with NO_3_^−^-N (Table 3).

**Table 2:** Correlations between soil properties and microbial community traits at days 30 and 60.

Variables	SOC	TN	TP	DOC	DON	NH_4_^+^-N	NO_3_^−^-N	SAP	pH	α-B	α-F	PC1_B	PC2_B	PC1_F	PC2_F
SOC	1														
TN	0.45*	1													
TP	0.77*	0.72*	1												
DOC	0.39*	0.15	0.14	1											
DON	−0.07	−0.16	0.00	0.01	1										
NH_4_^+^-N	0.49*	−0.07	0.33	0.23	0.27	1									
NO_3_^−^-N	0.56*	0.15	0.47*	0.15	−0.19	0.32	1								
SAP	0.77*	0.72*	1.00*	0.14	0.00	0.33	0.47*	1							
pH	0.60*	0.03	0.35	0.43*	−0.10	0.46*	0.41*	0.35	1						
α-B	−0.35	−0.08	−0.23	−0.30	0.07	−0.36	−0.19	−0.23	−0.40*	1					
α-F	0.60*	−0.02	0.28	0.33	−0.22	0.44*	0.53*	0.28	0.67*	−0.22	1				
PC1_B	0.13	−0.25	−0.17	0.20	−0.51*	−0.18	0.04	−0.17	0.24	0.02	0.33	1			
PC2_B	0.53*	0.12	0.47*	0.45*	0.04	0.41*	0.47*	0.47*	0.61*	−0.09	0.48*	−0.02	1		
PC1_F	−0.30	0.18	−0.07	−0.19	−0.11	−0.60*	−0.33	−0.07	−0.31	0.54*	−0.22	0.16	−0.2	1	
PC2_F	−0.30	−0.07	−0.23	−0.15	0.11	−0.09	−0.41*	−0.23	−0.54*	0.09	−0.72*	−0.09	−0.35	−0.14	1

An asterisk (*) indicates a significant correlation (*p* < 0.05). SOC, soil organic carbon; TN, total nitrogen; TP, total phosphorus; DOC, dissolved organic carbon; DON, dissolved organic nitrogen; NH₄^+^-N, ammonium nitrogen; NO₃^−^-N, nitrate nitrogen; SAP, soil available phosphorus; α-B, bacterial alpha diversity; α-F, fungal alpha diversity; PC1_B, PCoA1 of bacterial community; PC2_B, PCoA2 of bacterial community; PC1_F, PCoA1 of fungal community; PC2_F, PCoA2 of fungal community.

**Table 3:** Correlations between soil properties and microbial community traits at day 120.

Variables	SOC	TN	TP	DOC	DON	NH_4_^+^-N	NO_3_^−^-N	SAP	pH	α-B	α-F	PC1_B	PC2_B	PC1_F	PC2_F
SOC	1														
TN	0.31	1													
TP	0.21	0.74*	1												
DOC	0.07	0.48	0.50	1											
DON	0.12	0.27	0.26	0.418	1										
NH_4_^+^-N	0.50	−0.03	−0.08	−0.30	−0.22	1									
NO_3_^−^-N	0.48	0.33	0.35	0.43	0.79*	−0.125	1								
SAP	0.21	0.74*	1.00*	0.50	0.26	−0.08	0.35	1							
pH	0.96*	0.24	0.12	0.05	0.25	0.56*	0.53*	0.12	1						
α-B	−0.47	0.05	−0.08	−0.13	0.03	−0.32	−0.21	−0.08	−0.42	1					
α-F	0.67*	0.17	−0.17	−0.30	−0.03	0.56*	0.12	−0.17	0.63*	−0.16	1				
PC1_B	−0.04	0.17	−0.004	0.08	−0.21	−0.46	−0.03	−0.004	−0.07	0.35	−0.1	1			
PC2_B	−0.87*	−0.13	−0.15	−0.09	−0.10	−0.38	−0.54*	−0.15	−0.86*	0.62*	−0.47	−0.04	1		
PC1_F	−0.74*	−0.17	−0.02	0.18	−0.08	−0.43	−0.36	−0.021	−0.71*	0.50	−0.77*	0.11	0.69*	1	
PC2_F	0.04	0.02	0.05	0.27	0.41	−0.33	0.56*	0.05	0.15	−0.06	−0.27	0.33	−0.36	0.09	1

An asterisk (*) indicates a significant correlation (p < 0.05). SOC, soil organic carbon; TN, total nitrogen; TP, total phosphorus; DOC, dissolved organic carbon; DON, dissolved organic nitrogen; NH₄^+^-N, ammonium nitrogen; NO₃^−^-N, nitrate nitrogen; SAP, soil available phosphorus; α-B, bacterial alpha diversity; α-F, fungal alpha diversity; PC1_B, PCoA1 of bacterial community; PC2_B, PCoA2 of bacterial community; PC1_F, PCoA1 of fungal community; PC2_F, PCoA2 of fungal community.

Mantel test results indicated that both fungal and bacterial community structures were significantly associated with their respective ɑ-diversity metrics (*p* < 0.05; Supplementary Figure S3), suggesting that variations in community composition were, to some extent, linked to changes in within-community diversity. Spearman’s correlation analysis further revealed that fungal ɑ-diversity was positively correlated with biochar properties, including specific surface area, ash content, total carbon, total nitrogen, and soil pH. These results indicate that biochar physicochemical characteristics were closely associated with shifts in fungal ɑ-diversity (*p* < 0.05; Supplementary Figure S3), whereas their relationships with overall community structure were comparatively weaker.

**Figure 5:**
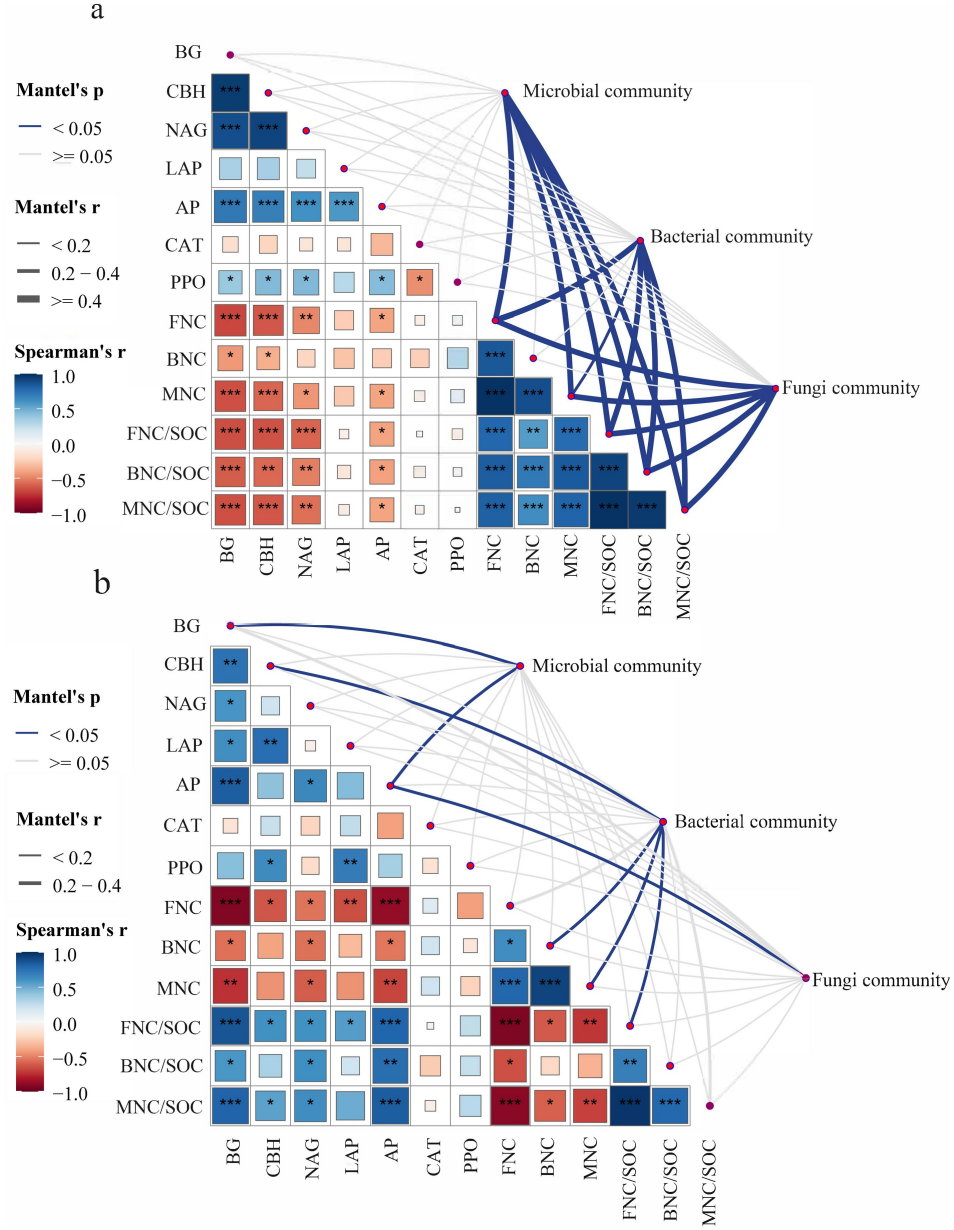
Heatmap of Mantel test correlations showing the relationships between microbial community structure and key soil variables. The analysis assesses the relationship between fungal and bacterial community composition (rows) and various metrics of microbial necromass and enzyme activity (columns). The data is presented for two distinct periods **(a)** Short-term (days 30 and 60 pooled) and **(b)** long-term (day 120). The color of each cell represents the strength and direction of the correlation, with red indicating a positive relationship and blue indicating a negative relationship. The numbers within the cells are Mantel’s *r* correlation coefficients. Statistical significance is denoted by asterisks: * *p* < 0.05, ** *p* < 0.01, *** *p* < 0.001. FNC, fungal necromass carbon; BNC, bacterial necromass carbon; SOC, soil organic carbon; FNC/SOC, contribution of fungal necromass to SOC; BNC/SOC, contribution of bacterial necromass to SOC; BG, β-1,4-glucosidase; CBH, cellobiohydrolase; NAG, N-acetyl-β-D-glucosaminidase; LAP, leucine-ɑ-aminopeptidase; AP, acid phosphatase; CAT, catalase; PPO, polyphenol oxidase.

**Figure 6:**
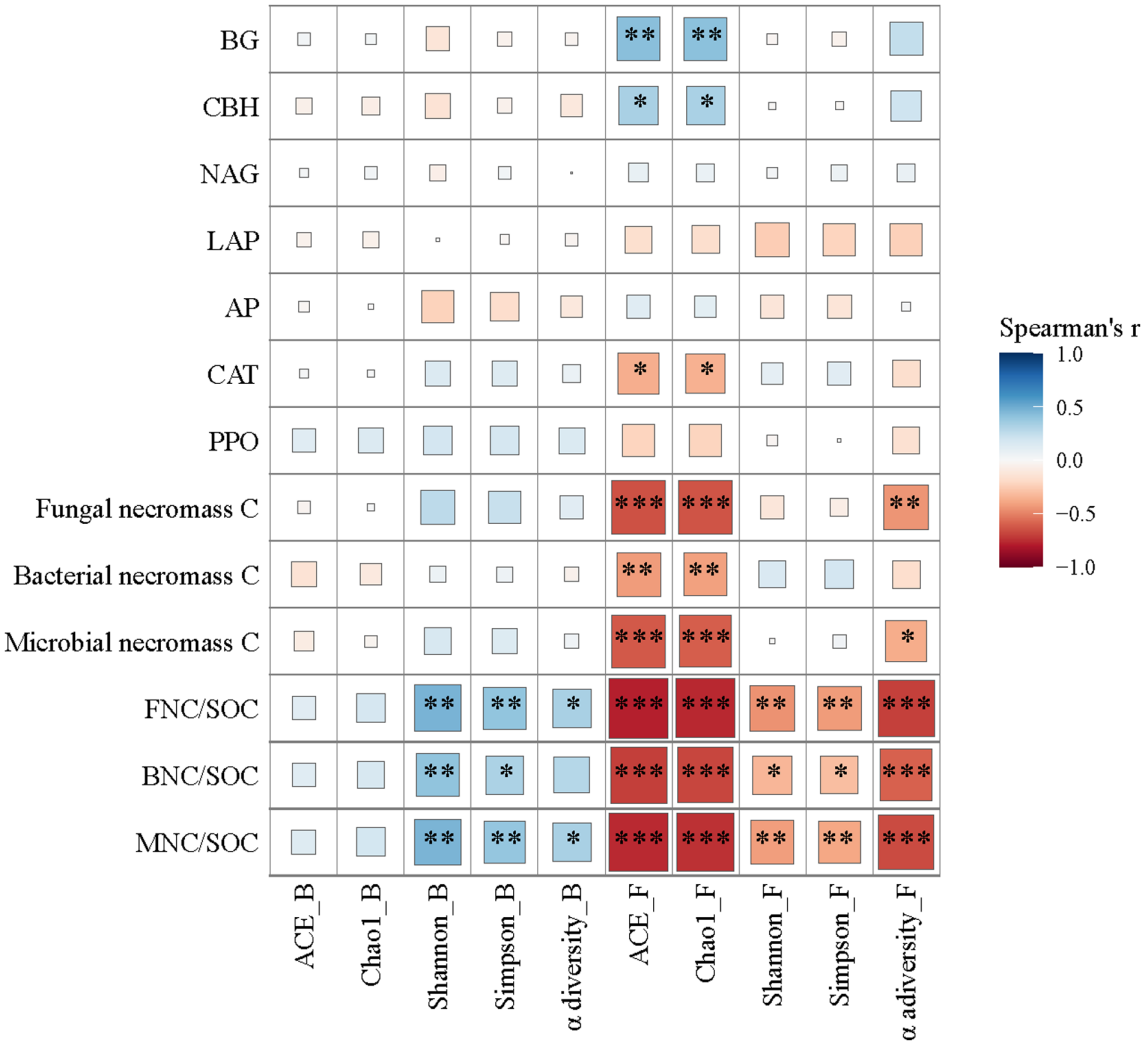
Spearman correlation analysis between extracellular enzyme activities, microbial necromass carbon pools, and microbial diversity indices. Extracellular enzyme activities include β-1,4-glucosidase (BG), cellobiohydrolase (CBH), β-1,4-N-acetylglucosaminidase (NAG), leucine aminopeptidase (LAP), phosphatase (AP), catalase (CAT), and polyphenol oxidase (PPO). Microbial necromass variables include fungal necromass carbon (FNC), bacterial necromass carbon (BNC), total microbial necromass carbon (MNC), and their relative contributions to SOC (FNC/SOC, BNC/SOC, and MNC/SOC). Microbial diversity indices comprise ACE, Chao1, Shannon, Simpson, and *α*-diversity for both bacteria (B) and fungi (F). Color intensity represents the strength and direction of Spearman’s correlation coefficients (*r*), with blue indicating positive correlations and red indicating negative correlations. Asterisks denote significance levels (* *p* < 0.05; ** *p* < 0.01; *** *p* < 0.001).

Mantel test results showed that on days 30 and 60, the community composition of fungi was a predictor of fungal necromass carbon and its contribution to SOC, but the bacterial community composition was not correlated with bacterial necromass carbon. Enzyme activities were not correlated with the microbial community. Fungal necromass carbon, FNC/SOC, and BNC/SOC were negatively correlated with BG, CBH, NAG, and AP. Bacterial necromass carbon was negatively correlated with BG and CBH. On day 120, the community composition of fungi was a predictor of AP, and the bacterial community composition was a predictor of CBH and bacterial necromass carbon. Fungal necromass carbon was negatively, but FNC/SOC was positively, correlated with BG, CBH, NAG, LAP, and AP. Bacterial necromass carbon was negatively, but BNC/SOC was positively, correlated with BG, NAG, and AP (Figure 5). Spearman’s correlation analysis revealed that both fungal community diversity were significantly associated with extracellular enzyme activities and microbial necromass carbon pools (*p* < 0.05; Figure 6). The fungal *α*-diversity indices showed significant correlations with enzymes involved in carbon and nitrogen cycling. Notably, fungal α diversity exhibited consistent and significant negative correlations with microbial necromass carbon (including BNC and FNC), and their proportional contributions to soil organic carbon. In contrast, bacterial diversity indices showed positive consistent relationships with necromass carbon pools (*p* < 0.05; Figure 6).

**Figure 7:**
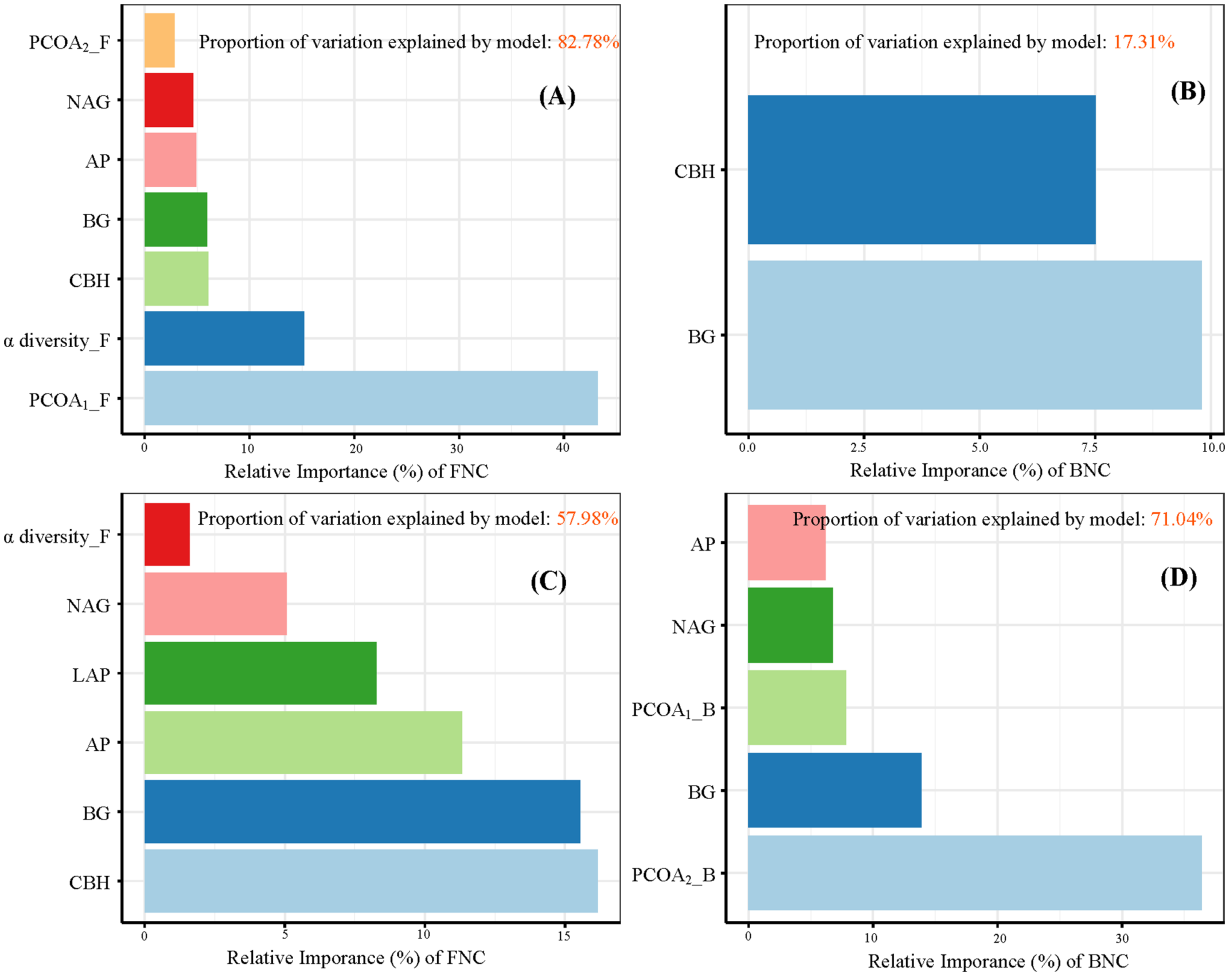
Relative importance of different microbial and enzymatic variables in predicting necromass carbon, determined by dominance analysis. The bars represent the percentage contribution of each predictor variable to the model’s total explained variance (R^2^). The analysis was conducted for two time periods: short-term (data pooled from days 30 and 60) and long-term (day 120). **(A)** Fungal necromass carbon (FNC) in the short term (Total R^2^ = 57.88%). **(B)** Bacterial necromass carbon (BNC) in the short term (Total R^2^ = 41.83%). **(C)** Fungal necromass carbon (FNC) in the long term. **(D)** Bacterial necromass carbon (BNC) in the long term. For the long-term models (C and D), the overall model fit (Total R^2^) was 70.94% for both. BG, β-1,4-glucosidase; CBH, cellobiohydrolase; NAG, N-acetyl-β-D-glucosaminidase; LAP, leucine-ɑ-aminopeptidase; AP, acid phosphatase; F_αdiversity, α-diversity of fungi; PCOA1_B, PCoA1 of bacterial community; PCOA2_B, PCoA2 of bacterial community; PCOA1_F, PCoA1 of fungal community; PCOA2_F, PCoA2 of fungal community.

The relative importance analysis revealed a strong fit when integrating the most influential factors among various microbial traits, with a modeled R^2^ value of 82.78 and 17.31% for fungal and bacterial necromass C on days 30 and 60, 57.98 and 71.04% for fungal and bacterial necromass C on day 120 (Figure 7).

## 4 Discussion

### 4.1 The response of microbial necromass carbon to biochar and its pyrolysis temperature varied with the duration of the experiment

At days 30 and 60, biochar amendment consistently reduced fungal and bacterial necromass carbon relative to the control. This reduction was mechanistically driven by pronounced shifts in microbial community composition, which were closely constrained by temperature-dependent changes in biochar physicochemical properties. Specifically, biochar addition led to a decline in *Actinobacteriota* and Basidiomycota—taxa associated with the production of recalcitrant, chemically resistant biomass—while promoting the dominance of *Ascomycota*, a fast-growing, copiotrophic fungal phylum (Treseder and Lennon, 2015). These results suggest a fundamental transition in community life-history strategies: from the accumulation of persistent residues (*Basidiomycota*/*Actinobacteriota* dominant) to rapid biomass turnover and resource acquisition (*Ascomycota* dominant).

Critically, this taxonomic restructuring coincided with increased fungal ɑ-diversity. Contrary to the common assumption that diversity enhances carbon stabilization via biomass accumulation (Wagg et al., 2014; Delgado-Baquerizo et al., 2016), our results revealed a strong negative correlation between fungal ɑ-diversity and necromass carbon pools. This apparent paradox suggests that in the biochar-amended environment, higher diversity functioned as a catalyst for metabolic turnover rather than residue preservation. We propose that biochar-induced pH neutralization and habitat heterogeneity acted as primary environmental filters, selecting for a phylogenetically diverse fungal community possessing a broader enzymatic repertoire (Rousk et al., 2010). Under conditions of intensified interspecific competition, this functionally diverse community likely maximized resource extraction efficiency (Maynard et al., 2017), thereby accelerating the enzymatic breakdown of vulnerable microbial residues rather than allowing them to accumulate. This interpretation is supported by the strong positive association between ɑ-diversity and extracellular enzyme activities (e.g., BG and CBH), which provided the biological potential for rapid necromass decomposition (Sinsabaugh et al., 2008; Liu et al., 2024).

This enzymatic potential was translated into actual necromass loss through metabolic feedbacks associated with nutrient mining and priming effects. The input of labile biochar-C created a stoichiometric imbalance (high C, limiting N), which triggered the “nutrient mining” mechanism: microorganisms intensified the enzymatic decomposition of N-rich microbial residues to acquire limiting nitrogen (Zhang M. et al., 2021). Simultaneously, the addition of utilizable carbon induced positive priming (Chen Y. et al., 2023), stimulating the degradation of recalcitrant organic matter (including native MNC) (Craine et al., 2007), while reducing the metabolic need to synthesize new structural necromass via the microbial carbon pump (Liang et al., 2017). Thus, the initial suppression of necromass carbon resulted from the coupling of a more efficient, diverse community (the potential) with strong resource-driven decomposition triggers (the driver).

The magnitude of these effects was temperature-dependent and primarily mediated by fungal communities, which are generally more sensitive than bacteria to nutrient stoichiometry in subtropical soils (Wang et al., 2021). The reduction in necromass carbon was most pronounced in lower-temperature biochar treatments, where higher contents of labile C and available nutrients maximized microbial stimulation. Conversely, high-temperature biochars (e.g., 650 °C), characterized by recalcitrant C and reduced nutrient availability, likely induced weaker priming and catalytic effects, thereby mitigating the net loss of necromass carbon (Guo et al., 2020).

By day 120, however, the trend reversed, with biochar increasing microbial necromass carbon regardless of pyrolysis temperature. This shift reflects the transient nature of biochar’s initial stimulatory effects. As labile nutrients and readily oxidizable C fractions were depleted via plant uptake and leaching(Chen Y. et al., 2023), the metabolic triggers for priming and nutrient mining subsided, as evidenced by the neutral responses of enzyme activities and inorganic N levels at day 120. Furthermore, the progressive formation of soil aggregates likely provided physical protection for microbial necromass against enzymatic attack (Yang et al., 2022). Consequently, as the intense initial turnover phase waned, the system shifted back toward net accumulation of microbial residues.

### 4.2 The response of microbial necromass carbon/SOC to biochar and its pyrolysis temperature varied with the duration of the experiment

At days 30 and 60, biochar significantly decreased the ratio of bacterial and fungal necromass carbon to SOC, consistent with global patterns (*p* < 0.05). On one hand, biochar reduced bacterial and fungal necromass carbon content mediated by soil nutrients and microorganisms, as described above. Additionally, the higher bacterial and fungal necromass carbon to SOC under BC_650_ treatment compared to other biochar types could be attributed to higher pyrolysis temperatures resulting in fewer available nutrients and thus less necromass carbon loss caused by the weaker decomposition, as discussed above. On the other hand, biochar addition can enhance SOC by promoting the accumulation of plant-derived carbon (Weng et al., 2017; Chen et al., 2024). These factors collectively decrease the contribution of microbial necromass carbon to SOC.

At day 120, the negative effect of biochar on the ratio of bacterial necromass carbon to SOC vanished, but the ratio of fungal necromass carbon to SOC under biochar treatments remained lower than CK. This might be explained by the asymmetric positive effect of biochar on fungal and bacterial necromass carbon. It is speculated that, in the short term, biochar increased the proportion of micro-aggregates, which preferentially favored the accumulation of bacterial necromass carbon (Han et al., 2021; Xu et al., 2022). Therefore, the positive effect of biochar on bacterial necromass carbon could be larger than on fungal necromass carbon. Additionally, the biochar pyrolyzed at the lowest temperature increased FNC/SOC but not fungal necromass carbon compared with BC_550_ and BC_650_. This might be due to asymmetric changes in necromass carbon and SOC (Qian et al., 2023) and the weaker promotion of low-temperature biochar on SOC (Supplementary Table S1).

### 4.3 Methodological considerations and implications for future studies

While this study provides mechanistic insights into the temporal dynamics of microbial necromass carbon under biochar amendment, several methodological considerations should be acknowledged.

First, regarding plant–microbe interactions, although sorghum was grown in all treatments to maintain a rhizosphere environment, plant biomass, root traits, and nutrient uptake were not quantified, as our primary objective was to elucidate the mechanisms of soil microbial necromass stabilization. Plant growth can strongly influence microbial necromass dynamics by altering rhizodeposition, which supplies fresh carbon to soil microorganisms, and by competing with microbes for limiting nutrients (Fan et al., 2025; Wan et al., 2025). Consequently, biochar-induced changes in plant performance may have indirectly influenced microbial activity and MNC formation. For instance, enhanced plant nutrient uptake in biochar-amended soils could have intensified microbial nitrogen limitation, potentially reinforcing the nutrient mining and priming mechanisms proposed in this study. Therefore, while the observed patterns primarily reflect microbially mediated processes, indirect plant-mediated effects cannot be completely excluded and should be explicitly addressed in future integrative studies that simultaneously quantify plant and soil microbial responses.

Second, regarding the biochar application rate, we acknowledge that the relatively high biochar addition rate used in this experiment (approximately 2–5%, w/w) exceeds those typically applied under field agronomic conditions. However, such application rates have been widely employed in pot and incubation experiments to amplify biochar-induced biogeochemical responses and disentangle underlying microbial mechanisms, as documented in previous meta-analyses and controlled studies (Cayuela et al., 2014; Wang J. et al., 2015). In this context, the objective of the present study was to identify process-level responses—particularly the links among microbial diversity, enzymatic activity, and necromass turnover—rather than to directly simulate field-scale management practices. Nonetheless, extrapolation to agricultural systems should be made with caution. Future long-term field experiments using agronomically realistic biochar application rates are required to assess whether the mechanisms identified here persist under complex field conditions and contribute to soil carbon stabilization under climate change scenarios.

Third, while we did not directly measure microbial carbon use efficiency (CUE) via isotopic tracing, the integration of amino sugar biomarkers (representing net necromass accumulation) with extracellular enzyme activities and microbial community profiles provides a robust proxy for inferring microbial metabolic strategies. The observed shifts in fungal/bacterial ratios and enzymatic stoichiometry effectively reflect the balance between microbial anabolism and catabolism driving MNC dynamics.

## 5 Conclusion

Vinasse biochar amendment elicited a biphasic impact on soil MNC, characterized by short-term suppression via nutrient mining and priming, followed by long-term accumulation. Crucially, high-temperature biochars mitigated initial carbon losses by limiting labile substrates. Mechanistically, we found that biochar-induced fungal diversity initially acted as a catalyst for decomposition rather than stabilization. However, by day 120, the system transitioned to net accumulation across all treatments. Therefore, optimizing pyrolysis temperature is vital for balancing the trade-off between transient metabolic priming and long-term necromass retention, offering a refined strategy for enhancing soil carbon sequestration.

## Data Availability

The datasets presented in this study can be found in online repositories. The names of the repository/repositories and accession number(s) can be found below: NCBI Sequence Read Archive (SRA) (BioProject accession number: PRJNA1473403; SRA accession number: SRP705921).
