# Supplementary figures and images for: The response of microbial necromass C and its contribution to SOC to vinasse biochar based on a pot experiment
Source: Front Microbiol. 2026 Jun 17;17:1737822. doi: 10.3389/fmicb.2026.1737822 (PMC13319103; doi:10.3389/fmicb.2026.1737822)

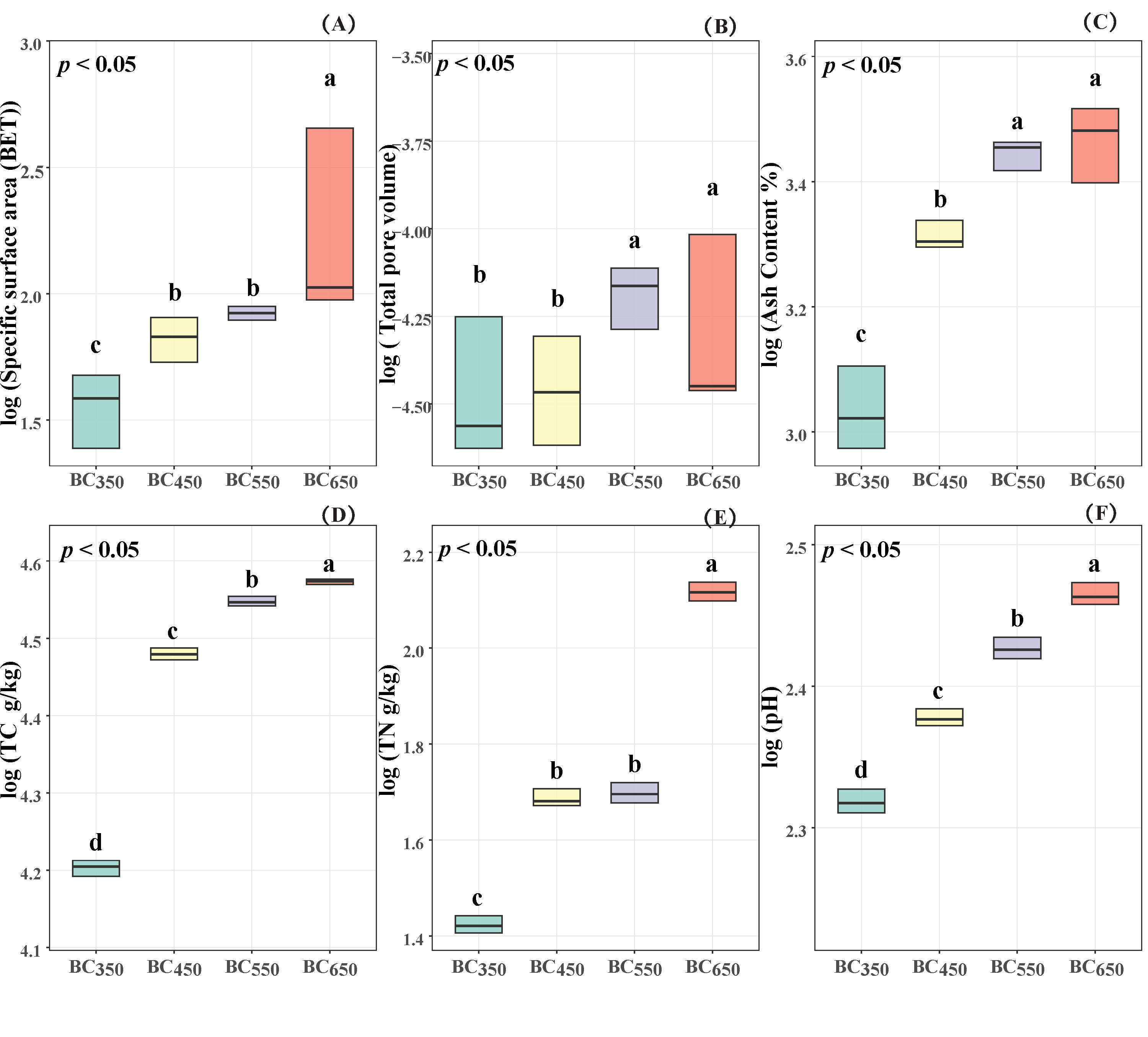

Supplement: Supplementary file 1 [file Image_1.TIF]

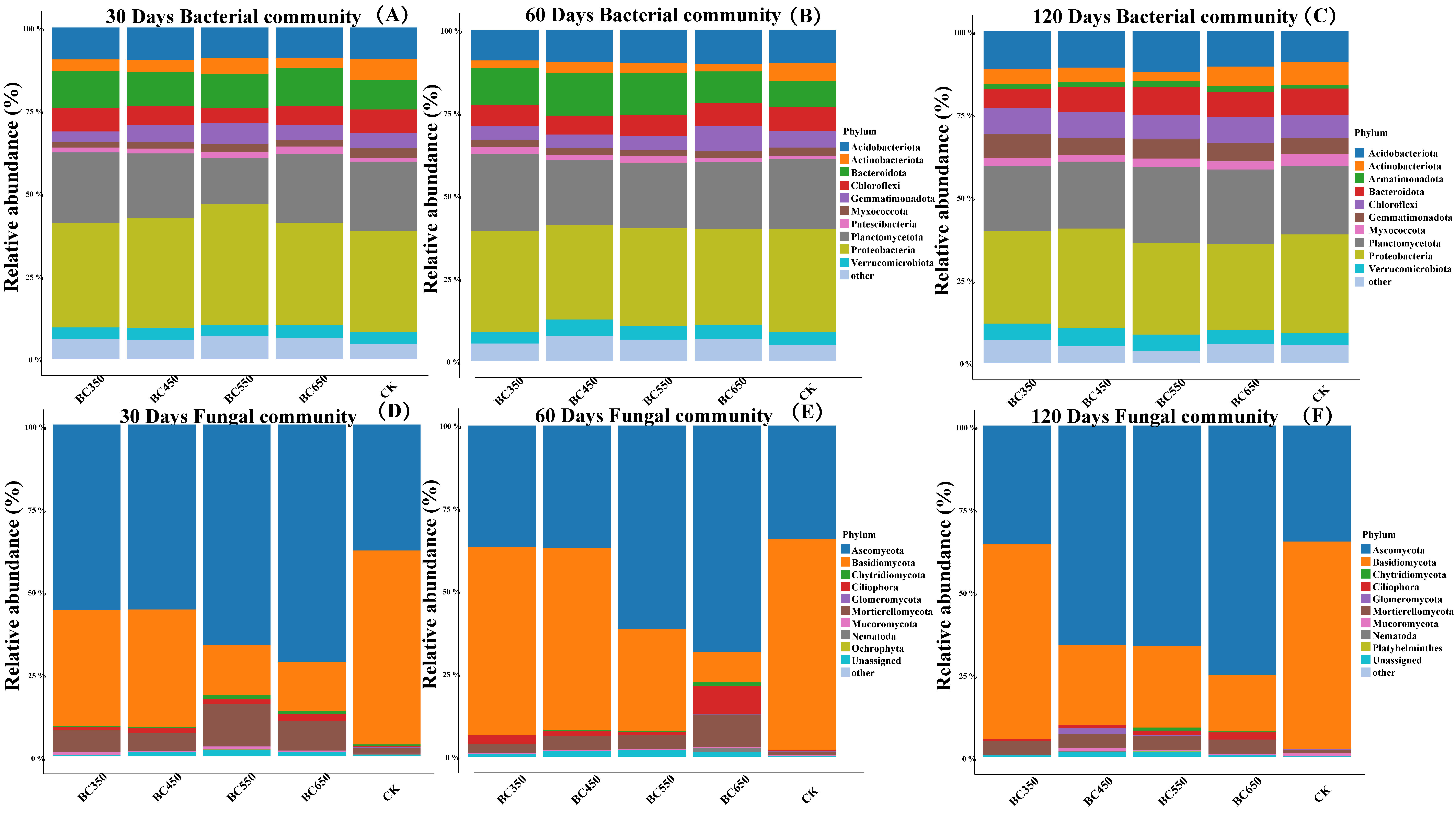

Supplement: Supplementary file 2 [file Image_2.TIF]

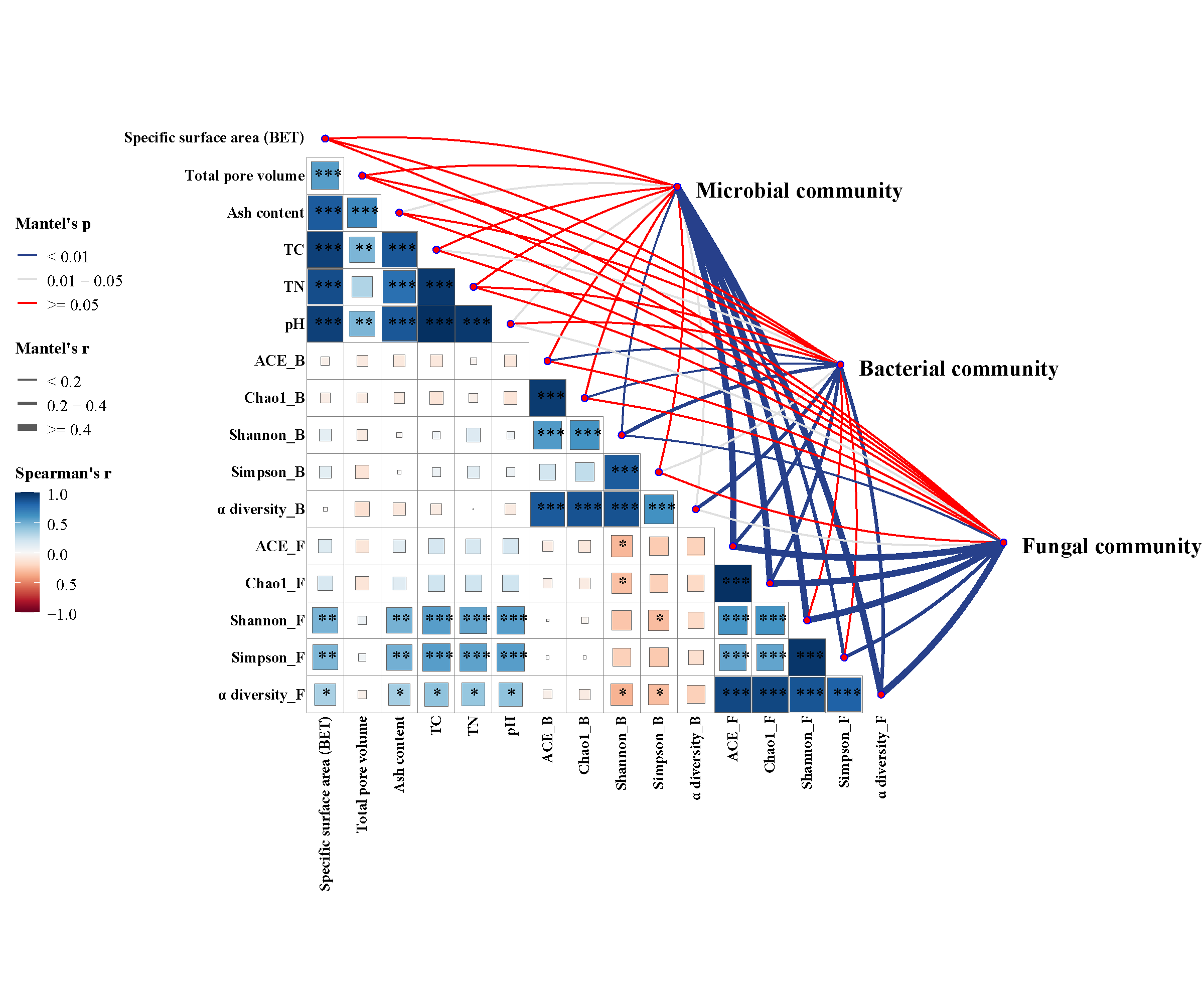

Supplement: Supplementary file 3 [file Image_3.TIF]
