# Supplementary material for: The response of microbial necromass C and its contribution to SOC to vinasse biochar based on a pot experiment
Source: Front Microbiol. 2026 Jun 17;17:1737822. doi: 10.3389/fmicb.2026.1737822 (PMC13319103; doi:10.3389/fmicb.2026.1737822)
Supplement: Supplementary file 4 [file Table_1.DOCX]

Table S1 The influences of all treatments on soil properties.

| Soil properties | Treatment | Day 30 | Day 60 | Day 120 |
| --- | --- | --- | --- | --- |
| SOC(g/kg) | CK | **22.09±1.10 B** | **16.62±0.67 D** | **25.84±2.92 D** |
|  | BC_350_ | **67.29±2.77 A** | **42.68±2.39 C** | **45.56±0.90 C** |
|  | BC_450_ | **63.89±12.41 A** | **48.95±0.72 B** | **52.06±1.98 BC** |
|  | BC_550_ | **63.48±4.01 A** | **53.93±1.97 B** | **57.71±1.21AB** |
|  | BC_650_ | **52.53±7.42 A** | **63.21±3.56 A** | **63.35±3.36A** |
| TN(g/kg) | CK | 1.63±0.03 AB | 1.38±0.10 A | 1.65±0.03 A |
|  | BC_350_ | 2.17±0.34 A | 1.27±0.08 A | 1.44±0.09 A |
|  | BC_450_ | 1.82±0.28 AB | 1.34±0.20 A | 1.72±0.25 A |
|  | BC_550_ | 1.51±0.14AB | 1.81±0.22 A | 1.52±0.19 A |
|  | BC_650_ | 1.26±0.06 B | 1.59±0.12 A | 1.99±0.28 A |
| TP(g/kg) | CK | 0.44±0.01 B | 0.36±0.01 B | 0.49±0.04 A |
|  | BC_350_ | 0.63±0.01 A | 0.41±0.03 B | 0.46±0.03 A |
|  | BC_450_ | 0.55±0.09 AB | 0.50±0.11 AB | 0.58±0.14 A |
|  | BC_550_ | 0.48±0.04 AB | 0.52±0.04 AB | 0.49±0.03 A |
|  | BC_650_ | 0.42±0.03 B | 0.62±0.05 A | 0.50±0.03 A |
| DOC(g/kg) | CK | **21.07±4.03 B** | 60.83±4.06 AB | 49.93±3.45 A |
|  | BC_350_ | **71.26±9.23 A** | 49.72±5.80 B | 49.84±6.13 A |
|  | BC_450_ | **54.13±8.82 A** | 55.12±7.41 AB | 42.26±9.265 A |
|  | BC_550_ | **63.70±15.56 A** | 69.38±3.83 A | 74.15±27.03 A |
|  | BC_650_ | **66.15±6.28 A** | 64.07±4.23 AB | 48.18±8.96 A |
| DON(g/kg) | CK | 45.27±14.75 A | 17.36±0.01 B | 18.08±0.29 A |
|  | BC_350_ | 25.76±6.81 A | 21.40±1.27 AB | 20.83±3.36 A |
|  | BC_450_ | 19.79±1.77 A | 26.95±1.53 A | 49.22±22.32 A |
|  | BC_550_ | 19.15±1.48 A | 19.42±2.33 AB | 26.79±7.95 A |
|  | BC_650_ | 26.19±6.52 A | 21.03±3.80 AB | 30.48±10.92 A |
| NH_4_^+^ -N(g/kg) | CK | **0.62±0.05 B** | **0.60±0.03 B** | 0.60±0.05 A |
|  | BC_350_ | **2.95±0.13 A** | **2.61±0.30 A** | 0.78±0.09 A |
|  | BC_450_ | **2.89±0.04 A** | **3.58±0.21 A** | 1.21±0.46 A |
|  | BC_550_ | **2.87±0.23 A** | **2.75±0.41 A** | 0.70±0.02 A |
|  | BC_650_ | **3.03±0.09 A** | **2.87±0.14 A** | 0.91±0.07 A |
| NO_3_^-^-N(g/kg) | CK | 0.28±0.03 A | **0.30±0.04 B** | 0.31±0.04 A |
|  | BC_350_ | 0.48±0.02 A | **0.53±0.06 A** | 0.33±0.14 A |
|  | BC_450_ | 0.50±0.06 A | **0.54±0.04 A** | 0.47±0.12 A |
|  | BC_550_ | 0.43±0.14 A | **0.50±0.08 A** | 0.68±0.16 A |
|  | BC_650_ | 0.46±0.04 A | **0.58±0.04 A** | 0.60±0.16 A |
| SAP(g/kg) | CK | 44.22±1.15 B | 36.42±0.84 B | 48.59±4.17 A |
|  | BC_350_ | 62.66±0.51 A | 40.70±2.86 B | 45.83±3.10 A |
|  | BC_450_ | 55.49±9.16 AB | 49.64±11.26 AB | 58.05±13.61 A |
|  | BC_550_ | 48.18±4.19 AB | 51.98±4.35 AB | 48.60±3.08 A |
|  | BC_650_ | 41.87±3.19 B | 61.867±4.97A | 49.63±2.74 A |
| pH | CK | **6.04±0.02 D** | **6.02±0.03 D** | **6.01±0.01 E** |
|  | BC_350_ | **6.34±0.06 C** | **6.24±0.02 C** | **6.18±0.01 D** |
|  | BC_450_ | **6.36±0.01 C** | **6.30±0.02 C** | **6.32±0.04 C** |
|  | BC_550_ | **6.60±0.04 B** | **6.58±0.03 B** | **6.60±0.01 B** |
|  | BC_650_ | **6.88±0.03 A** | **6.86±0.03 A** | **6.89±0.02 A** |

Note: The bold font indicates that this soil property varies significantly across all treatments at this time (n = 3). Different capital letters indicate the significant difference (P < 0.05) between different treatments. The value represent mean±SE. Note: SOC, soil organic carbon. TN, total nitrogen. TP, total phosphorus. DOC, dissolved organic carbon. DON, Dissolved organic nitrogen. NH_4_^+^-N, ammonium nitrogen. NO_3_^-^-N, nitrate nitrogen. SAP, soil available phosphorus.
